# Spatially Explicit Data: Stewardship and Ethical Challenges in Science

**DOI:** 10.1371/journal.pbio.1001634

**Published:** 2013-09-03

**Authors:** Joel Hartter, Sadie J. Ryan, Catrina A. MacKenzie, John N. Parker, Carly A. Strasser

**Affiliations:** 1 Department of Geography, University of New Hampshire, Durham, New Hampshire, United States of America; 2 Department of Environmental and Forest Biology, SUNY College of Environmental Science and Forestry, Syracuse, New York, United States of America; 3 Center for Global Health and Translational Science, Department of Immunology and Microbiology, Upstate Medical University, Syracuse, New York, United States of America; 4 Faculty of Life Sciences, University of KwaZulu-Natal, Pietermaritzburg, South Africa; 5 Department of Geography, McGill University, Montreal, Quebec, Canada; 6 Barrett, the Honors College at Arizona State University, Arizona State University, Tempe, Arizona, United States of America; 7 California Digital Library, Oakland, California, United States of America

## Abstract

Scholarly communication is at an unprecedented turning point created in part by the increasing saliency of data stewardship and data sharing. Formal data management plans represent a new emphasis in research, enabling access to data at higher volumes and more quickly, and the potential for replication and augmentation of existing research. Data sharing has recently transformed the practice, scope, content, and applicability of research in several disciplines, in particular in relation to spatially specific data. This lends exciting potentiality, but the most effective ways in which to implement such changes, particularly for disciplines involving human subjects and other sensitive information, demand consideration. Data management plans, stewardship, and sharing, impart distinctive technical, sociological, and ethical challenges that remain to be adequately identified and remedied. Here, we consider these and propose potential solutions for their amelioration.

## Abstract 2

Sharing spatially specific data, which includes the characteristics and behaviors of individuals, households, or communities in geographical space, raises distinct technical and ethical challenges.

## Introduction

Datasets and accompanying metadata are an important currency of scientific and intellectual advancement, deserving the same attention, planning, and scrutiny that research dollars receive. The move towards digital data is ubiquitous across disciplines [1]–[5]: earth scientists use satellite data to understand global patterns; ecologists use GPS tagging of mammals to understand migration paths; biomedical researchers produce and consume record amounts of clinical and genetic information; and social scientists are inundated with social media data. These data must be synthesized and analyzed to conceptualize, comprehend, and solve real-world problems [6].

The digital nature of data means more data more quickly. This “data deluge” has been explored in academic literature [1],[5],[7],[8] and major media including *The Economist* [9] and *The New York Times* [10]. Among the most pressing problems associated with it is good data stewardship—the ability to effectively and efficiently record, curate, and facilitate access to large volumes of data. For in actuality, data are seldom shared, re-used, or preserved [11]–[13], resulting in inefficient use of research dollars, missed opportunities to exploit prior investment, and overall loss for the scholarly community [14]. The development of good data stewardship techniques, software, and education lags behind the data deluge.

In February 2011, the US National Science Foundation (NSF) [15] prescribed that a two-page data management plan must accompany all research proposals. The National Science Board's Data Policies Task Force informs this requirement:

“Progress in science and engineering has always been dependent on the collection of data through observation, experimentation, and more recently, computation. A core expectation of the scientific process is the documentation and sharing of results along with the underlying data and methodology, thereby allowing others to verify data, reproduce results, validate interpretations, and build upon previous work. (p.17)”

To improve data stewardship for publicly funded projects, several US governmental funders (e.g., NSF, NOAA, USDA, EPA, DOD, NASA, NIH, CDC, DOE) require data management plans (DMPs) for all proposed research [16], and some journals request that supporting data be made available upon publication [17]. Data sharing policies are also in place for the Research Councils UK, a consortium of seven research councils (http://www.rcuk.ac.uk/research/Pages/DataPolicy.aspx), and the Digital Curation Centre (DCC) lists the specific DMP requirements by funder (http://www.dcc.ac.uk/resources/data-management-plans/funders-requirements). Canada's NSERC specifies data management requirements for grants through SSHRC and CRIC (http://www.nserc-crsng.gc.ca/Professors-Professeurs/FinancialAdminGuide-GuideAdminFinancier/Responsibilities-Responsabilites_eng.asp), as does the Australian National Data Service (ANDS) (http://www.ands.org.au/resource/data-management-planning.html). Institutions interested in protecting their investments increasingly look to libraries and information professionals to collaborate with scientists [18]; researchers, in turn, demand properly managed data from their colleagues [13].

Researchers are ethically obliged to be good data stewards to advance scientific knowledge, but those working with human subjects must also protect participant confidentiality [19]. Previously, meeting these ethical obligations fell to the individual researcher (or team) and was managed in an *ad hoc* manner. Human subjects research renders careful data stewardship more than a matter of scientific rigor—it requires ensuring confidentiality while providing sufficient information for validation, reproducibility, reuse, and reporting. The need for rigor and data acquisition must be balanced against the ethical treatment of participants.

**Figure 1:**
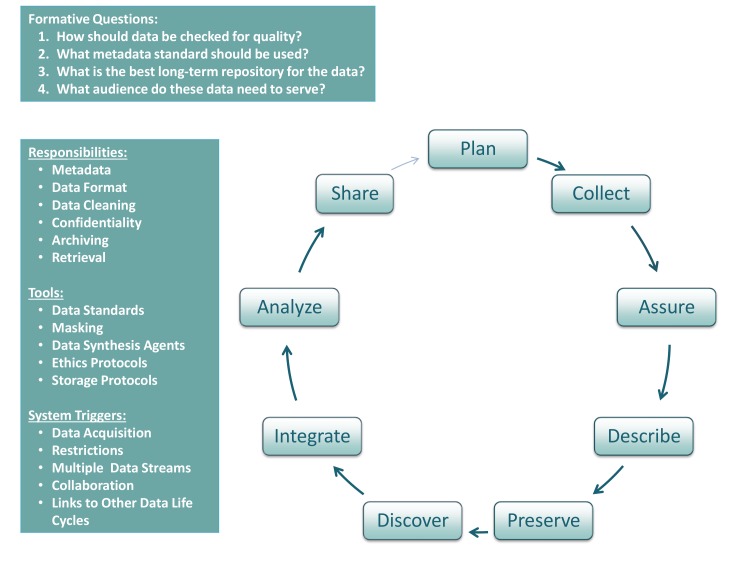
The life cycle of data: the steps needed to responsibly collect, record, store, and steward data. We illustrate the steps needed to responsibly collect, record, store, and steward data, from collection planning and design to sharing endpoints. The formative questions are a basic guide to researchers at the outset of a project, to shape the design of a robust dataset with an extended life. The responsibilities and tools are similarly guidance for consideration; the system triggers are a non-comprehensive list of when researchers might find themselves stepping into the cycle.

Data management plans require careful consideration of accessibility and data sharing; imparting challenges that have yet to be adequately identified and addressed. Data sharing has transformed the practice, scope, content, and applicability of scientific research [20],[21], and as calls for data stewardship increase, researchers need to consider how to most effectively comply. We examine these inherent technical, socio-cultural, and ethical challenges and propose some means for solving them. Figure 1 summarizes the data life cycle in context of this discussion of stewardship and sharing.

### Technical Challenges

The technical challenges of sharing scientific data abound, and are amplified in certain disciplines. For example environmental (including social) data are “messy” in ways that are not the case across much of the physical sciences [22],[23]. Datasets are often small, heterogeneous, collected via a wide array of methods, stored in a wide variety of formats, and analyzed using a plurality of methods and techniques. The variability of research approaches engaging human subjects (ranging from observations to attitudinal and network surveys to the social scientific methods of interviewing and ethnographic observations) and data types (ranging from numeric data points to photos, video, interview transcriptions, ethnographic field notes, audio and visual recordings, and medical records) challenge the ability to store, retrieve, combine, use, and meaningfully re-analyze data [24],[25].

Data sharing requires substantial time, energy, and technical capacity to organize, store, and preserve data and make them widely accessible [26], while potentially masking or securing sensitive or confidential information. It requires designing and implementing rigorous metadata standards, and the creation of flexible, intuitive databases [27]. Simultaneously, there arises the real danger of data misinterpretation due to insufficient metadata standards [28],[29]. Such technical challenges are heightened in remote locations or in situations wherein the necessary capital—i.e., technicians and data managers familiar with metadata language, programs, and standards—is unavailable, or where discrepancies are created by discipline-specific norms. Ironically, these are precisely the locations and conditions in which much field research for conservation biology or emerging infectious disease, for example, takes place.

### Socio-Cultural Challenges

Data sharing requires shifting from a research culture predicated on perpetual proprietary control over data to one that promotes scientific openness, and which values analysis and synthesis of secondary data [20],[21],[30]–[32]. In science it is common to secret data and dole out findings selectively in accordance with strategic publication practices (e.g., [33],[34]). Competition can create anxiety about being “scooped” by colleagues; data sharing raises the particularly vexing specter of being beaten to the punch with one's own data [35],[36]. Further, the benefits and dangers of data sharing are distributed unequally; e.g., scholars working in sensitive, high-profile, highly politicized systems are at greater risk of being scooped. Unequal data sharing risks also emerge for scholars with limited funding, working far from the academic mainstream or at smaller institutions. Data sharing is moreover challenged by varying disciplinary practices and expectations, and by diverse organizational and institutional cultures. Interdisciplinary research data gatherers and those with whom data are shared can have divergent epistemological assumptions, professional mandates, and reward systems [37]–[39], and legal and ethical standards for data sharing and protecting research subjects.

The study of “social-spatial linkage”—the characteristics and behaviors of individuals, households, or communities in geographical space—represents an important scientific advancement [40], but including human subjects also introduces the risk of confidentiality breach [41]. Global Positioning System (GPS) technology allows spatially explicit longitudinal studies [42], and increasing satellite and aerial imagery, coupled with GPS and radio-frequency identification tags, now provides voluminous information on the activities of people, animals, cars, etc. within dynamic landscapes. Geospatial technologies such as unmanned aerial vehicles, Google Earth, Google Maps, Wikimapia, and Open Street Maps offer unprecedented access to place-specific data and surveillance capabilities [43]. While these data are helpful for making maps, they can introduce complications. For example, conservation biology focuses on rare species, habitats, and resources, but identifying their locations with high-resolution geospatial data may render them vulnerable to abuse and extraction. Additionally, while social science data are integral to conservation [44],[45], their inclusion adds related ethical challenges.

Traditionally, geographic information mainly existed as maps and atlases produced by mapping authorities, agencies, and corporations, subsequently dispersed to users. Maps emphasized static attributes; now, input from users is being used for emergencies and everyday use [46]. Locational crowdsourcing or volunteered geographic information (VGI) is an exciting new area of data generation and geographic information delivery [47], wherein citizen volunteers contribute geographic data and geo-tagged photos. An important advance in data collection and delivery [47]–[50], this is also one of the greatest ethical challenges because it can provide near real-time, dynamic snapshots of one-the-ground conditions [51],[52]. Within the data deluge, geographic information is more readily accessible, created and distributed by a network of observers. Protocols and institutions are needed to ensure that the result is reliable, useful, and ethical [48]–[50],[53].

### Ethical Challenges

Increased data-sharing requirements pose potentially significant challenges to researchers since they must ensure their work meets the ethical standards of academia [54]. These standards require that research with human subjects respects individuals, commits to nondisclosure of participants, minimizes potential harm, ensures that the benefits and burdens of research be fairly distributed [19], and that subjects be informed of the full nature of the research so they can opt out of participation. Researchers' strategies for addressing these ethical standards must be clearly detailed when applying for ethics approval from Institutional Review Boards (IRBs) [55].

The primary benefit of capturing locational human subjects data (e.g., socio-economic conditions and demographics) is to support longitudinal research, help avoid over-researched locales, and capture locational effects (e.g., elevated lead levels [56]). The ability to identify and locate these study “spaces” requires even stricter data control to protect confidential information. New methods aggregate social data at larger scales or mask data locations, allowing data interpolation using less distinct spatial patterns. New spatially explicit IRB standards and virtual data management institutions are being piloted to improve privacy protection [57]–[60]. Researchers new to human subjects research may not be sensitive to the ethical restrictions of human data, or know that sharing spatially explicit data can breach confidentiality commitments. Additionally, research subjects may not want their responses to be traceable for fear of retribution, stigmatization, or prosecution. Maintaining confidentiality protects participants while promoting willingness to participate in future studies. Yet alteration of spatially specific data to protect confidentiality can undermine data quality and reliability. For instance, the United States Forest Service Forest Inventory and Analysis National Program does not divulge the locations of the thousands of research plots throughout the US, hindering site-specific longitudinal studies. This suggests that better and more sensitive data ambiguation techniques are needed.

Some IRBs now require that spatially explicit social data be kept confidential or that anyone with data access be made aware of their ethical obligations and added to ethics approval (e.g., [61]). For instance, an integrated study linking spatially explicit social data to other datasets required the originating IRB to approve all future uses of these social data, creating logistical challenges for data sharing and collaborative research. Beyond confidentiality, sharing social data requires that subsequent researchers understand how participant harm was managed by the original researcher. For instance, research into illegal activities oblige the researcher to protect subjects from retribution by aggregating data or masking locations [62]. Since datasets often contain general socio-economic data, other researchers unaware of the original use of these data could publish maps permitting identification of participants. There may also be risks created by publishing seemingly benign data—consider the implications of household tribal affiliation data when ethnic unrest erupts. Hopefully, socio-ecological data sharing will not face the extremes of protection required for spatially explicit medical data, but social researchers are still bound by the ethics of their discipline, and human subject data sharing requires developing methods to retain confidentiality and protect participants from harm [63].

### Conclusions and Recommendations

Strategies for meeting the aforementioned challenges include: (1) establishing standards and norms of practice; (2) outlining governance structures to support human subjects-related data; and (3) enacting culture change towards better data stewardship.

#### Establishing standards and norms

Data sharing challenges are dominated by issues of variable methods, data, storage systems, and workflows. While unlikely that researchers will adhere to a limited set of research systems and methods, we should begin building on existing methods to facilitate synthesis. Tools such as social media, crowdsourcing, blogs, and wikis have enormous potential for fostering communication and collaboration around particular methods, analyses, or data types. Furthermore, durable and robust methods for synthesizing and sharing heterogeneous data have been pioneered at high-profile research centers (e.g., National Center for Ecological Analysis and Synthesis and the National Center for Evolutionary Synthesis). Such skill sets, statistical techniques, software packages, and data curation protocols should be widely disseminated and training programs instantiated.

#### Outlining governance structures

Protecting human subject confidentiality, ensuring safety, and preventing data misuse are increasingly complicated as data become more widely available. Data governance structures have not yet caught up to the pace of technology, thus many established laws (notably copyright) are inappropriate for digital datasets. IRB approval for human subjects research is similarly lagging. We must therefore define new rules and regulations tailored to digital data, with careful consideration for social-spatial data. Furthermore, researchers must be trained in data stewardship and responsible development of IRB protocols and data management plans.

#### Enacting culture change

We must move towards a norm of openness and sharing. Openly shared datasets require careful documentation with clearly outlined policies and procedures for appropriate use. Researchers must become much better data stewards, with an in-depth understanding of metadata, best practices for data organization, and plans for archiving and preserving data. Importantly, so must institutions; data stewardship takes time and resources, and researchers cannot simply be expected to be data stewards without sufficient resources and support. There are many data standards (e.g., Darwin Core, The Conservation Measurement Partnership [64]) and tools (see e.g., dmptool.org; ecoinformatics.org; dataup.cdlib.org) available for facilitating good data stewardship. Emerging workflow systems (e.g., Kepler and Taverna) hold the promise of automated analytical workflows that can be shared, reused, and archived alongside datasets.

The technical, socio-cultural, and ethical challenges associated with data stewardship mentioned here are not the only ones. New mandates are created by funders as the culture of data stewardship evolves, and new challenges will arise as data volume and precision increases. Coping with interdisciplinary differences will require cross-disciplinary graduate training (e.g., NSF IGERT programs) to lower cultural and epistemic barriers between disciplines. Differing organizational mandates and reward systems are more difficult to manage, but could be better accomplished by making researchers aware of these differences and working to find the “sweet spot” wherein collaborators' organizational mandates, reward systems and research interests converge [39]. The importance of sharing both data and findings to build new knowledge and advance science are paramount. We therefore challenge researchers, practitioners, and policy-makers to devise the appropriate means, guidelines, and tools to responsibly manage the rising tide of the data deluge.
